# The role of microglia in adult hippocampal neurogenesis

**DOI:** 10.3389/fncel.2013.00229

**Published:** 2013-11-22

**Authors:** Carmelina Gemma, Adam D. Bachstetter

**Affiliations:** ^1^Department of Anesthesiology and Pain Medicine, University of WashingtonSeattle, WA, USA; ^2^Sanders-Brown Center on Aging, University of KentuckyLexington, KY, USA

**Keywords:** microglia, CX3CR1, neurogenesis, fractalkine, chemokines

## Abstract

Our view of microglia has dramatically changed in the last decade. From cells being “silent” in the healthy brain, microglia have emerged to be actively involved in several brain physiological functions including adult hippocampal neurogenesis, and cognitive and behavioral function. In light of recent discoveries revealing a role of microglia as important effectors of neuronal circuit reorganization, considerable attention has been focused on how microglia and hippocampal neurogenesis could be an interdependent phenomenon. In this review the role of microglia in the adult hippocampal neurogenesis under physiological condition is discussed.

## Adult Neurogenesis: the generation of new neurons in the mature CNS

Neurogenesis occurs throughout life in the adult mammalian brain including humans (Eriksson et al., 1998; Roy et al., 2000; Wang et al., 2011). In rodents, neurogenesis occurs predominantly in the subgranular zone (SGZ) of the dentate gyrus in the hippocampal formation, and in the subventricular zone (SVZ) of the lateral ventricle (Gage, 2000). The process of generating new neurons consists of four phases: proliferation, migration, differentiation, and survival. Studies over the last decade have elegantly described each step in the neurogenic process (for review see Ming and Song, 2011). Adult hippocampal neurogenesis originates from a population of proliferating radial and non-radial precursor cells located in the SGZ, which give rise to neural progenitor cells (NPCs), which in turn generate neuroblasts (Ehninger and Kempermann, 2008). Immature neurons migrate into the granule cell layer and differentiate into dentate granule cell in the hippocampus. Although NPCs proliferation generates a vast number of neurons only a very small proportion survives for long period of time (Kempermann et al., 2003). Indeed, most of the newly born cells are eliminated by apoptosis during first few days following birth. The cells that do survive for the first two weeks are then stable and integrated into the network of the dentate gyrus throughout life. After this time point, only very small changes in cell number occur. There are only two critical periods for neural progeny survival: (1) during transition from amplifying neuroprogenitors to neuroblast (Platel et al., 2010; Sierra et al., 2010); and (2) during the integration stage of the immature neurons (Tashiro et al., 2006; Mouret et al., 2008). By two months the surviving neurons receive input from other neurons (van et al., 2002; Piatti et al., 2006), with some forming functional synapses, and possessing electrophysiological properties indistinguishable from those of mature neurons (Ge et al., 2008).

## Microglia heterogeneity

 Microglia are recognized as the resident brain immune cells and have important roles in the healthy central nervous system (CNS). For example as the brain’s tissue macrophage, a primary function of microglia is to phagocytose dying cells and cellular debris silently (i.e., without producing inflammation). However, upon a pathologic insult, such as infection or brain injury, microglia respond rapidly. Part of this reactive response includes a morphological change in appearance. In the healthy CNS, microglia have highly *ramified* morphology with thin processes, which dynamically move in the brain parenchyma in what has been called a surveillance state (Nimmerjahn et al., 2005). We define microglia in this surveillance state as *ramified microglia*. In contrast, *reactive microglia*, (i.e., microglia that are no longer ramified microglia) can adopt a number of altered morphologies, including a hypertrophic cell with enlarged processes, or amoeboid macrophage like morphology.

## Microglia: role of ramified microglia in adult neurogenesis

Increasing evidence suggests that ramified microglia are an essential component of the neurogenic niche in the SGZ of the adult hippocampus. One of the critical roles of microglia in modulating hippocampal neurogenesis is pruning of newborn cells during the first critical period of survival. Sierra et al. (2010) demonstrated that ramified microglia have an important function in phagocytosis of apoptotic cells during the first days of their life. As the newborn cells are integrated into the existing circuits other cells become apoptotic. The apoptotic cells are subsequently removed by microglia in an immunologically silently process (i.e., without inflammation). In response to tissue damage, such as a traumatic brain injury, damage associated molecular patterns (DAMPs: e.g., Adenosine triphosphate (ATP), DNA) are released and cause microglia to become activated towards a proinflammatory state. In the context of neurogenesis, the dying cells undergo a process of programmed cell death, and no DAMPs are released. As opposed to the phagocytosis by ameboid microglia observed during trauma or neurodegeneration, phagocytosis of apoptotic cells during neurogenesis is performed by ramified microglia. Ramified microglia remove apoptotic neurons by a special modification of the microglial process, which form phagocytic pouches that engulf the apoptotic cells. The phagocytic pouches, occurs independent from the cell body, in terminal or en passant branches, as opposed to engulfment of the soma by ameboid microglia. Furthermore, phagocytosis of apoptotic neurons by ramified microglia is highly efficient as demonstrated by the high proportion of apoptotic cells engulfed by ramified microglia, the proportion of microglia engaged in the engulfing process, and the time to completely eliminate apoptotic cells (Sierra et al., 2010).

## Microglia provide trophic support for adult hippocampal neurogenesis

**Figure 1:**
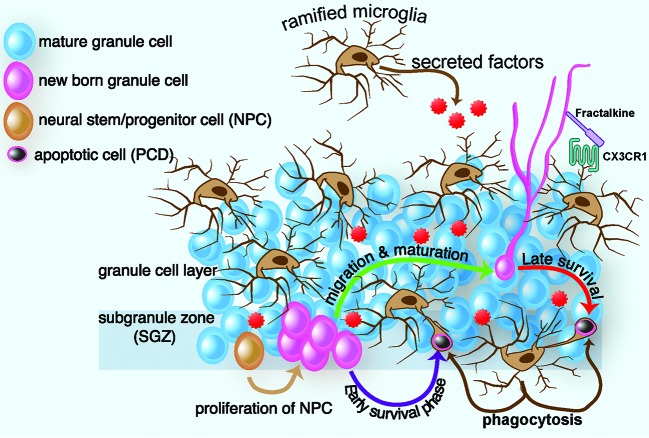
**Schematic diagram of ramified microglia and their effect on adult hippocampal neurogenesis.** In intact brain, microglia regulate several steps of adult hippocampal neurogenesis. In the SGZ, progenitor cells migrate to the granule cell layer and differentiate into a neuronal phenotype, with most NPCs dying in the first few days of life. Within two months, the surviving neurons receive input, form functional synapses with their target cells, and exhibit electrophysiological properties indistinguishable from those of mature neurons. In intact brain, ramified microglia eliminate apoptotic newborn cells during the first few days of their life by phagocytosis. This phagocytosis occurs by a special modification of the microglial processes, which form phagocytic pouches that engulf the apoptotic cells. Microglia can also affect proliferation, differentiation, and survival, through the secretion of neurotrophic factors. Finally microglia communicate with nearby neurons through the CX3CR1/CX3CL1 signaling. Interactions between CX3CL1 and CX3CR1 contribute to the ability of microglia to maintain a surveillant/ramified phenotype. Disruption of this signaling results in a change in microglia phenotype and function, which leads to decreased hippocampal neurogenesis.

 The role of microglia in neurogenesis is not limited to removal of cells. Evidence suggests that microglia secrete factors that can influence proliferation, differentiation into neurons or glia, and survival of the newborn cell. How could microglia-derived factors influence neurogenesis? Several different mechanisms have been proposed: (1) microglia could have a direct instructive role in dictating the commitment to a neuronal phenotype, (2) microglia could promote proliferation through secretion of neurotrophic factors, and (3) microglia could produce factors that regulate survival of neuronal cells. In favor of a direct instructive role of microglia to direct neuronal differentiation, *in vitro* studies demonstrate that microglia have the capacity to guide the differentiation of precursor cells isolated from embryonic brain as well as adult mouse neural precursor cells toward a neuronal phenotype (Aarum et al., 2003). In addition, precursor cell cultures grown in conditioned media from microglia cells contain a higher proportion of neurons than what would be expected from their spontaneous differentiation alone. Yet, microglia can affect proliferation and survival, in addition to neuron differentiation. For example, Morgan et al. (2004) further investigated the effect of non-stimulated microglia on neuronal proliferation and survival. The authors used microglia-conditioned medium collected from primary rat microglia to treat neurons *in vitro* for 7 days and found that the microglia-conditioned medium induced a 50% increase in neuronal survival compared to untreated neurons. A different study confirmed and extended these findings showing that addition of microglia-conditioned media in SVZ-derived culture increased neuroblast production (Walton et al., 2006). Furthermore, loss of inducible neurogenesis was paralleled by microglia depletion in proliferating culture. While a number of growth factors secreted by microglia could be responsible for such effect, evidence suggests that microglia are capable of producing growth factors, such as Insulin-like growth factor 1 (IGF-1) and Brain-derived neurotrophic factor (BDNF), which promote neurogenesis (Ziv and Schwartz, 2008). Figure 1 highlights the known ways microglia are involved in the neurogenic process.

In rodents, environmental enrichment has been one of the most clear and reproducible ways to stimulate adult neurogenesis (van et al., 1999; Inokuchi, 2011). Environmental enrichment has many beneficial effects on CNS function; intriguingly, some of the effects of environmental enrichment may be mediated by microglia. A recent finding showed that when microglia extracted from the hippocampus of runner mice were added to the hippocampal preparation of sedentary mice, the number of NPCs in the hippocampal culture of sedentary mice increased (Vukovic et al., 2012), suggesting that microglia are intrinsically altered or “primed” by the enriching experience. Indeed, following an enriched environment or physical activity, beneficial microglia increase, and this increase correlates with an increase in hippocampal neurogenesis (Ziv et al., 2006; Choi et al., 2008). However, other studies have shown no correlation or an inverse correlation in the role of microglia in neurogenesis stimulated by environmental enrichment (Gebara et al., 2013).

## Neuron–microglia dialogue in adult hippocampal neurogenesis

Until recently, neurons were believed to be submissive to the effects of microglia. However, a number of neuronal signals were found that can regulate microglia activation (Biber et al., 2007), suggesting a neuron-microglia dialogue. Indeed, neurons may also deliver signals that keep microglia in their surveillance mode indicating normal function. Under physiological conditions several neuron-mediated signals have an anti-inflammatory action at the level of the microglia. Cluster of differentiation (CD) 200 (also called OX2), CD47, CD55, CX3CL1 (fractalkine), are all neuro-immunoregulatory proteins constitutively expressed in healthy neurons with a cognate receptor on microglia (Kierdorf and Prinz, 2013). In the context of adult hippocampal neurogenesis, the question to be considered is whether neurons in the neurogenic niche communicate with microglia to regulate neurogenesis. A recent report suggests that the NPCs could secrete factors that regulate microglia function. Using an *in vitro* assay, the conditioned medium from NPCs caused microglia to increase in numbers, migrate to the site of injury, and to become phagocytic (Mosher et al., 2012). As previously mentioned, microglia can regulate neurogenesis at a number of steps in the neurogenic process. Therefore, a bidirectional regulation of neurons/neurogenesis and microglia might provide a means to fine tune the neurogenic process.

## Fractalkine/CX3CR1 as an example of neuron–microglia dialogue

As noted, *ramified* microglia dynamically move their processes within a volume of parenchyma and, in normal conditions, target synaptic structure (Wake et al., 2009; Tremblay et al., 2010). How do microglia know where to move their processes? What signals do microglia receive when they survey the neurons? Are there different signals for synaptic pruning or neuron pruning? Some of these questions are beginning to be answered, but this is an exciting area of microglia biology that is largely unexplored.

One of the best-characterized examples of a neuronal signal that regulates microglia function is the chemokine fractalkine. The anatomical expression of fractalkine on neurons and CXRCR1 on microglia led to the hypothesis of a unique signaling whereby neurons may maintain microglia in a surveillant/ramified state through a repressive fractalkine signal. Fractalkine is constitutively expressed at high levels on healthy neurons. The receptor for fractalkine, CX3CR1, is more highly expressed on microglia, than macrophages. Over the past decade numerous investigators have provided strong support that fractalkine does suppress microglia activation (Cardona et al., 2006; Ransohoff et al., 2007; Bhaskar et al., 2010; Lee et al., 2010).

Interestingly, data suggest that fractalkine signaling may be involved in neuron-microglia dialogue in the neurogenic niche that regulates neurogenesis. First it was shown by genetic deletion or pharmacological antagonism of CX3CR1 in young adult rats that CX3CR1 was important for the maintenance of hippocampal neurogenesis, as animals with decreased CX3CR1 have less neurogenesis (Bachstetter et al., 2011). Furthermore, levels of fractalkine, which are abundantly expressed in young healthy rodent brains, were decreased in aged rodents (Bachstetter et al., 2011). It was suggested that the decrease in fractalkine signaling may contribute to the increased neuroinflammation and decreased hippocampal neurogenesis seen in the aged rodent brain. To test this hypothesis, aged rats were administered fractalkine, which reversed the age-related decrease in hippocampal neurogenesis and restored microglia to a ramified morphology (Bachstetter et al., 2011).

Loss of fractalkine/CX3CR1 signaling in a non-disease model, not only affects neurogenesis, it can cause impairments in motor learning, cognitive function, and synaptic plasticity through increased microglia activation and inflammation in the CNS (Rogers et al., 2011). Impairment of long-term potentiation (LTP) and neurogenesis likely represent the mechanism responsible for the defect observed in hippocampal-dependent associative and spatial memory formation. However, multiple mechanisms could account for the impairment in cognitive function and synaptic plasticity observed in the CX3CR1-deficient mice. Studies on CX3CR1/fractalkine signaling and neuronal activity have produced discordant results. For example, exogenous application of fractalkine inhibits LTP and LTP impairment failed to occur in CX3CR1 deficient mice (Maggi et al., 2009). Differences in experimental protocols, animal age and gender, and housing conditions etc. could explain the discordance between studies.

As noted earlier, environmental enrichment, including exercise can stimulate neurogenesis. Recent data suggest that fractalkine/CX3CR1 signaling may be involved in the exercise-induced increase in hippocampal neurogenesis. Exercise reversed the age-related decline in fractalkine hippocampal levels and increased hippocampal neurogenesis (Vukovic et al., 2012). Furthermore, pharmacological antagonism of CX3CR1 prevented the increase in hippocampal neurogenesis (Vukovic et al., 2012). Therefore, the aforementioned results suggest that fractalkine signaling is a way microglia function could be tuned by neurons. A previous interesting study (Maggi et al., 2011) showed that absence of CX3CR1 in female mice leads to an increase in hippocampal plasticity and learning performance and a decrease in hippocampal-dependent response to environmental stimulation. In addition, deficiency of CX3CR1 in female mice blunted the positive effect of an enriched environment on neuronal plasticity. These controversial results are quite intriguing as they clearly show a gender difference effect of microglia in synaptic plasticity. Interesting, profound sex differences in the microglia colonization of the developing rodent brain were observed recently. At P4, male rats have significantly more microglia than females in many brain regions critical for cognition, learning, and memory including the hippocampus, parietal cortex, and amygdala (Schwarz and Bilbo, 2012). It would be interesting to know if a gender difference in microglia density exists during adulthood. Taken together these studies demonstrate that microglia through the CX3CR1 receptor play a physiological role in adult hippocampal neurogenesis and cognitive function.

## Conclusion

Microglia are an essential component of the neurogenic niche and emerging evidence shows new and fundamental roles for microglia in the control of neuronal proliferation, differentiation, and survival of newborn neurons into the existing neuronal circuitry. However the specific molecular and cellular mechanisms through which microglia regulate different stages of neurogenesis are only beginning to be explored. A deeper understanding of the physiological function of microglia in the different steps of adult neurogenesis is needed.

## Conflict of interest statement

The authors declare that the research was conducted in the absence of any commercial or financial relationships that could be construed as a potential conflict of interest.
